# Hierarchical LiFePO_4_ with a controllable growth of the (010) facet for lithium-ion batteries

**DOI:** 10.1038/srep02788

**Published:** 2013-09-27

**Authors:** Binbin Guo, Hongcheng Ruan, Cheng Zheng, Hailong Fei, Mingdeng Wei

**Affiliations:** 1 Institute of Advanced Energy Materials, Fuzhou University, Fuzhou, Fujian 350002, China

## Abstract

Hierarchically structured LiFePO_4_ was successfully synthesized by ionic liquid solvothermal method. These hierarchically structured LiFePO_4_ samples were constructed from nanostructured platelets with their (010) facets mainly exposed. To the best of our knowledge, facet control of a hierarchical LiFePO_4_ crystal has not been reported yet. Based on a series of experimental results, a tentative mechanism for the formation of these hierarchical structures was proposed. After these hierarchically structured LiFePO_4_ samples were coated with a thin carbon layer and used as cathode materials for lithium-ion batteries, they exhibited excellent high-rate discharge capability and cycling stability. For instance, a capacity of 95% can be maintained for the LiFePO_4_ sample at a rate as high as 20 C, even after 1000 cycles.

Rechargeable lithium-ion batteries (LIBs) are now considered as the next generation of power sources used in electric vehicles (EVs), hybrid vehicles (HEVs), and plug-in hybrid electric vehicles^1^,^2^,^3^. Since olivine-type LiFePO_4_ (LFP) was reported as a positive electrode material for LIBs by Padhi *et al.*^4^, it has been the subject of intense investigations for its potential as an energy storage material. Compared to cobalt-based electrode materials, LFP has the advantages of inherent merits including low toxicity, potential for low cost, long cycle ability and high safety^5^,^6^. Because of the slight change in unit cell parameters during the LiFePO_4_/FePO_4_ phase transition, the active material can be reversibly charged and discharged with a stable voltage profile at 3.45 V vs. Li^+^/Li. However, the poor conductively prevents its use on a large scale. To overcome these disadvantages, various approaches such as carbon coating^7^,^8^, conductive surface coating^9^, lattice doping^10^, and particle size narrowing^11^,^12^,^13^ have been adopted. Although these optimizing strategies can significantly enhance the material's electrochemical performance, major advances in preparation strategy and nanostructured design are still required to improve the properties of LFP-based LIB systems^14^. Despite the improved electrical conductivity of the powders after carbon coating, this method cannot solve the low intrinsic ionic conductivity of LFP^15^. Size reduction to nanoscale dimensions has been pointed out as one of the effective routes for solving the kinetic problems of LFP, but downsizing resulted low volumetric density are likely to influence commercial applications. The fabrication of hierarchical micro-nanostructured LFP has been considered as one of the promising ways to overcome these troubles^14^,^16^. This proposed structure has nano-sized primary structures to ensure high rate capability and micro-sized secondary structures to guarantee a high tap density^17^.

Recently, hierarchically structured LFP has been reported to facilitate the fast and efficient transport of mass and charge, and exhibited a better rate capability and considerable reversible capacity in comparison with the samples without hierarchical nanostructures^14^,^16^,^17^,^18^,^19^. Up to now, a simple synthetic route to fabricate hierarchically micro-nanostructured LFP has not been developed, and to the best of our knowledge, facet control of a hierarchical LFP crystal has not been reported yet. The orientation of LFP particles, which is strongly related to the kinetics of the lithium ion extraction/insertion process, is also an important factor that determines the electrochemical performance of LIBs^20^,^21^. This result was confirmed recently by research showing that lithium ion conductivity can be improved by tuning the particle size of LFP and by insuring a [010] orientation^22^,^23^,^24^. The Li^+^ ion diffusion takes place along the *b*-axis (010 direction) in the crystal structure of orthorhombic LFP (space group: Pnma) during the charge and discharge process^25^,^26^, with the charge transfer occurring mainly on the (010) facet^27^,^28^. Therefore, the developing an effective route to control both the hierarchical micro-nanostructure and the crystal facet of LFP is still a challenge.

Ionic liquid (IL) has high thermal stability, high solubility, negligible vapor pressure, and is environmentally friendly. Thus, it has attracted much attention in various fields of chemical synthesis, electrochemical applications and so forth^29^. According to report^30^, the size and morphology of LFP can be influenced significantly with the introduction of IL into the reaction system. When IL is used as a solvent rather than a stabilizer, the viscosity of IL slows down the ion diffusion rate, and thus prevents particles from growing. When using IL as a structural directing agent, the crystal orientation of LFP can be controlled. Because of the flexible nature of the cationic/anionic pairs, IL functionalized with different groups led to varying preferential orientation of crystal facet. Thus, IL can be used to control both particle size and the orientation of crystal growth.

In the present work, a simple one-step solvothermal route was first developed for synthesizing hierarchically structured LFP. This material exhibited a bow-like morphology with the (010) facet mainly exposed. The hierarchical LFP was used as an electrode for lithium ion intercalation and exhibited excellent high-rate performance and cycling stability.

## Results

### Structure of LiFePO_4_

**Figure 1:**
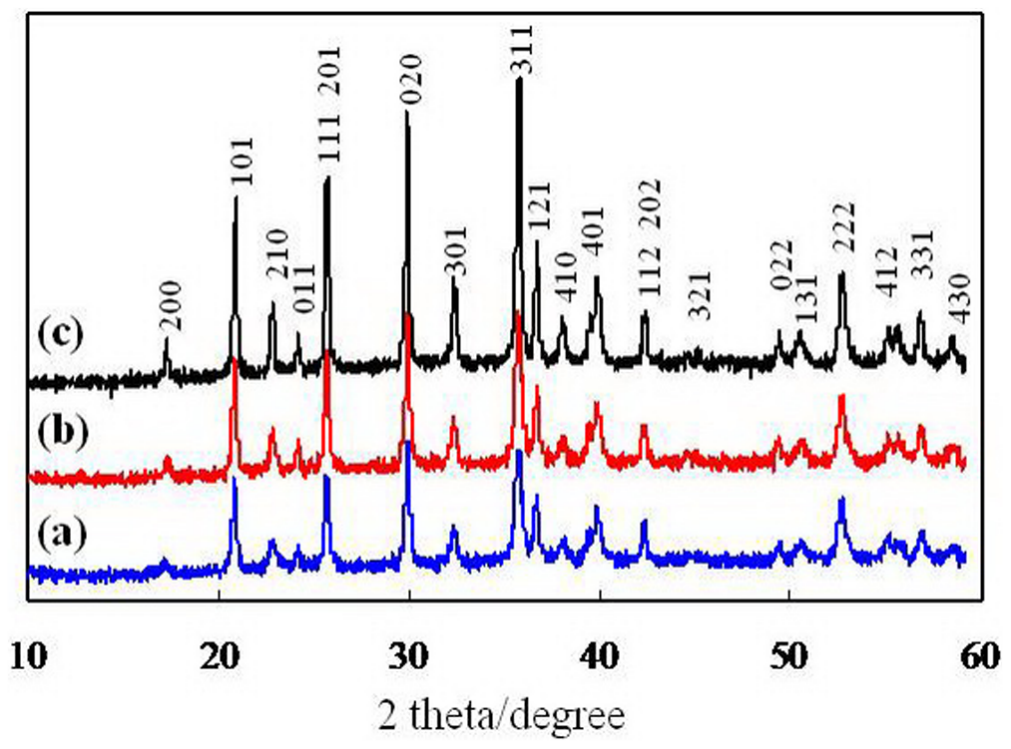
XRD patterns of the samples: (a) LFP-1, (b) LFP-2 and (c) LFP-3.

Fig. 1. shows X-ray (XRD) patterns of three LFP samples synthesized with different concentrations of the precursor (0.1, 0.2 and 0.4 mmol) and donated as LFP-1, LFP-2 and LFP-3. All three samples exhibited a pure phase of LFP with an olivine structure indexed to an orthorhombic Pnma space group (JCPDS 83-2092). The XRD pattern of LFP exhibited more narrow diffraction peaks and higher intensities when the concentration of the precursor was increased from 0.1 to 0.4 mmol. Of note, an important feature in XRD pattern of LFP was the peak intensity ratio of I{020}/I{200}. According to the report by Kanumara et al.^20^, they suggested that if the intensity of the (200) peak is greater than that of the (020) peak, a needle-shaped crystal was present, while a platelet-type structure was present if the intensity of (020) peak is greater than that of (200) peak. Therefore, three samples in the present work have a possibly plate-like morphology.

**Figure 2:**
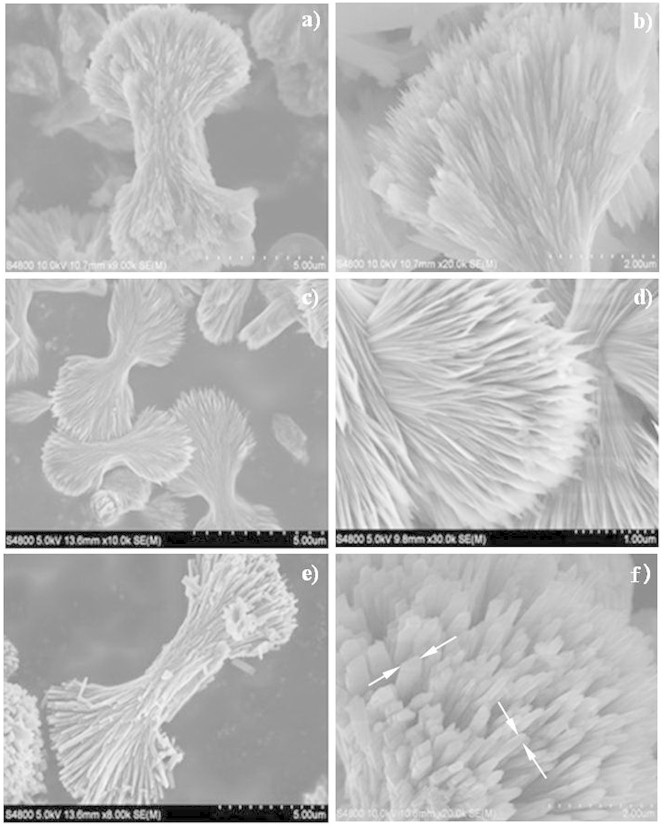
SEM images of the samples: (a, b) LFP-1, (c, d) LFP-2 and (e, f) LFP-3.

Fig. 2 shows the scanning electron microscopy (SEM) images of three LFP samples. The low-magnification SEM images in Fig. 2 (a, c, e) clearly show that the three samples all exhibited a similar bow-like morphology. Fig. 2 (b, d, f) reveals that the bow-like LFP was a hierarchical structure, constructed from platelet subunits. The lengths, widths, and thickness of these platelets varied with the concentrations of the precursor. The platelets of LFP-1 in Fig. 2b were too thin to measure their thickness and width from the SEM image. On the other hand, the thickness and width of platelets increased obviously after the concentration of the precursor was increased to 0.2 mmol (LFP-2), as depicted in Fig. 2d. When the concentration was further increased to 0.4 mmoL (LFP-3), the size of platelets increased significantly. As presented in Fig. 2f, the thickness and width of the platelets were 100–200 and 300–500 nm, respectively. These results indicate that the concentration of the precursor in the reaction system played a key role in the formation of hierarchically structured LFP.

**Figure 3:**
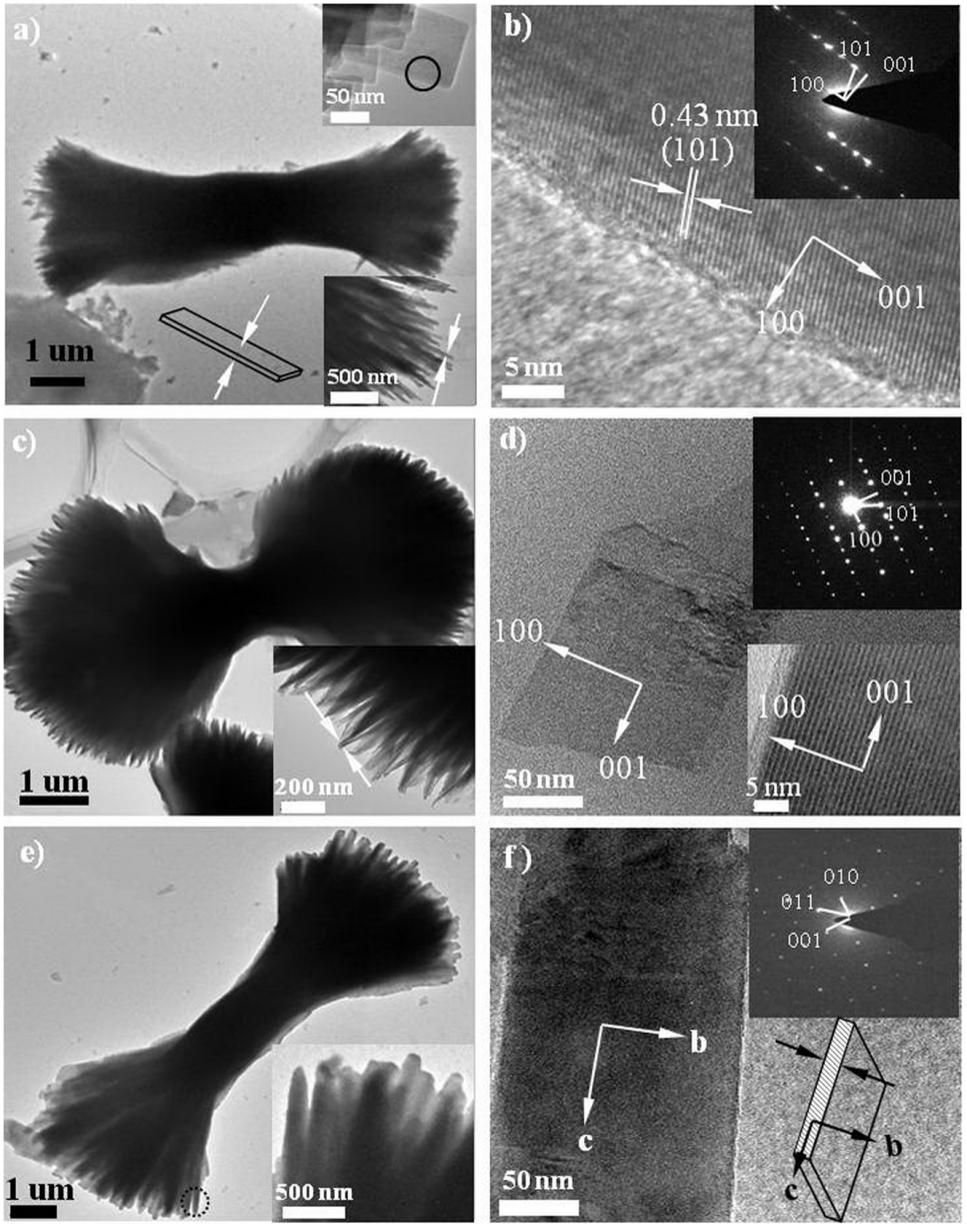
TEM images of the samples: (a, b) LFP-1, (c, d) LFP-2 and (e, f) LFP-3.

To confirm the morphology of LFP, transmission electron microscopy (TEM) images of the three samples were taken, as shown in Fig. 3. Fig. 3 (a, c, e) clearly shows a single bow-like LFP constructed from platelet subunits, confirming that the synthesized LFP has a hierarchical structure. As depicted in the insets of Fig. 3a, the thicknesses and widths of the platelets were found to be approximately 10–15 and 50–100 nm, respectively. The growth orientation was determined by indexing the high-resolution TEM (HRTEM) image and the corresponding selected-area electron diffraction (SAED). Fig. 3b shows an HRTEM image of a platelet subunit; the lattice fringe was 0.43 nm, corresponding to the interplanar spacing of {101} facets. The related SAED pattern in the inset of Fig. 3b shows that the platelet was highly crystallized, with the domain (010) facet exposed. This result demonstrates that the crystallized LFP platelets grown along the [101] direction agglomerated together to form a bow-like morphology via interconnection of {010} facets. Fig. 3c shows a LFP-2 sample that was grown with a 0.2 mmol of precursor. The micrograph shows that both ends of the LFP-2 bow were curled up, indicating that the concentration of precursor influenced the morphology of the finial LFP products. A single crumbled platelet was chosen and its HRTEM image was presented in Fig. 3d, revealing that the platelet was single crystalline, growing along the [101] direction, with (010) as the largest exposed facet. As depicted in Fig. 3e, the size of the platelets increased significantly with the increased precursor concentration to 0.4 mmol; their thicknesses were found to be approximately 100–200 nm, which was in agreement with the SEM results. Thus, the three LFP samples all had mainly exposed (010) facets, and grew along the [101] direction. These results are also in agreement with the XRD results in Fig. 1, in which the platelets exhibited strong (020) peak intensity.

### Growth mechanism

To shed light on the formation mechanism of the hierarchically structured LFP, a series of samples were synthesized with different reaction times, with results shown in Fig. S1. It can be seen from Fig. S1a that LFP was the main phase, while the impurities Li_3_PO_4_ (JCPDS 15-0760) and Fe_3_(PO_4_)_2_·8H_2_O (JCPDS 75-1186) were also detected at a short reaction times. With the reaction time was increased to 12 h, the impurities began to disappear, as shown in Fig. S1b. After the reaction time was further increased to more than 18 h, pure LFP was obtained as depicted in Fig. S1(c–d). At the same time, the diffraction peaks of LFP became narrower and stronger, indicating that the crystallinity of LFP was also improved.

SEM images of samples synthesized with different reaction times were also taken, as depicted in Fig. S2. It can be seen from Fig. S2a that, at low reaction times (6 h), some of the products exhibited spindle-like bulk structures, assembled by platelets. After the reaction time was increased to 12 h, the spindle-like bulk structures became exfoliated and the platelets began to curl up from the ends, as shown in Fig. S2b. With increasing time to 18 and 24 h, a product with a bow-like morphology was obtained, as shown in Fig. S2(c–d), indicating the exfoliated platelets curled up more in order to release the strain energy.

**Figure 4:**
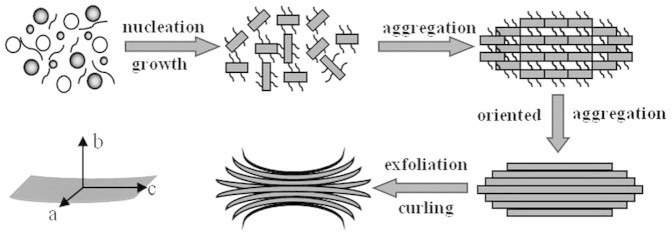
Schematic of a tentative mechanism for the formation of hierarchically structured LFP.

Based on these results, a possible mechanism for the formation of hierarchically structured LFP was proposed and depicted in Fig. 4. First, the mixing of reactants was dissolved in the IL solvent and the reaction was executed under solvothermal conditions. By increasing the reaction time, the nuclei of LFP were formed in reaction system. These nuclei led to growth, and platelets were formed. At the same time, a spindle-like bulk structure was formed through homoepitaxial aggregation of the platelets. After the reaction time was further increased, the spindle-like bulk continue grow. Driven by the minimization of the total energy of the system, the initially formed LiFePO_4_ nanoplates trended to assemble in edge-to-edge and layer-by-layer growth style based on the IL interaction. With increasing reaction time, the spindle-like bulk was gradually exfoliated from the ends. Subsequently, the formed platelets on both ends of the spindle-like bulk structure curled up to release high levels of stress^31^. As a result, the hierarchically structured LFP with a bow-like morphology was formed. In fact, the reports dealing with the formation of nanostructured materials starting from the exfoliation of the layered structure compounds or curling of sheets have been concerned^32^,^33^. On the other hand, IL played a key role for the formation of hierarchical structure. As well known, IL with a high viscosity would ensure a relatively slow condensation reaction between two crystal facets of LFP and further permit better crystal orientation alignment when aggregation occurs. At the same time, IL as a structural directing agent would lead to form a preferential orientation growth^30^. In fact, LFP with a similar morphology in the present of surfactant additives or polymeric has been widely investigated^34^,^35^.

### Electrochemical performance

**Figure 5:**
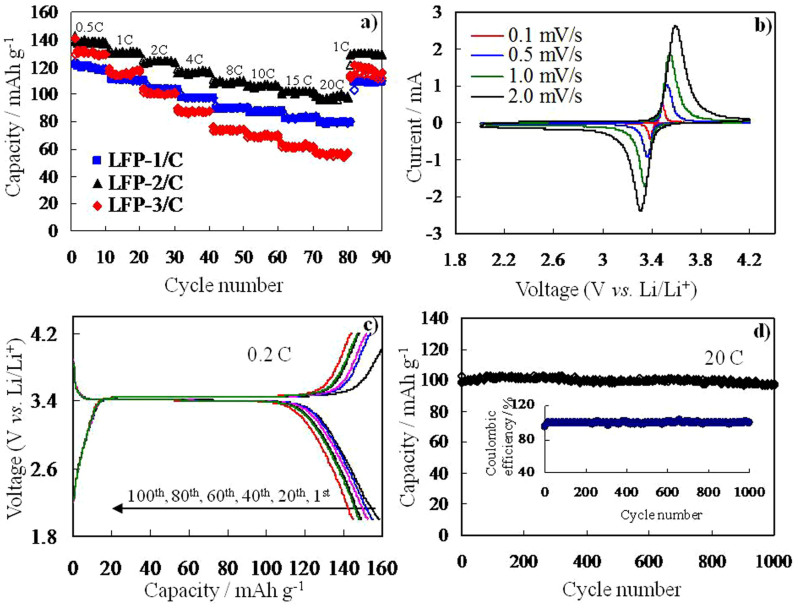
(a) Rate performance of hierarchical LFP/C composite at different current densities, and (b) CV curves, (c) charge-discharge profiles and (d) cycling performance of LFP-2/C composite. The inset: Coulombic efficiency.

To improve electron conduction, the hierarchically structured LFP samples were also coated with a thin layer of carbon, and their hierarchical structure can be maintained (Fig. S3). The three samples coated with a carbon layer are named LFP-1/C, LFP-2/C and LFP-3/C. The three samples were tested at different current densities in the voltage range of 2.0–4.2 V. Fig. 5 shows the electrochemical properties of the cells made with hierarchically structured LFP. It can be seen from Fig. 5a that all three samples all exhibited attractive rate capabilities and the remained stable when the rate was returned to 1 C. Among them, the LFP-2/C exhibited an attractive rate capability with initial discharge capacities of 139, 131, 123, 116, 108, 106, 100, and 98 mAh g^−1^ at 0.5, 1, 2, 4, 8, 10, 15, and 20 C, respectively. To estimate the effects of LFP with a bow-like structure and carbon coated on the surface of LFP, the electrochemical properties of uncoated LFP-2 were also shown in Fig. S4. Fig. 5b shows CV curves of the LFP-2/C sample at different scanning rates. The well-defined, sharp redox peaks in the range of 3.30–3.60 V should be attributed to the Fe^2+^/Fe^3+^ redox couple reaction, corresponding to lithium extraction and insertion in the LFP crystal structure. Even at a high scanning rate of 2.0 mV s^−1^, the sharp redox reaction peaks are maintained. Fig. 5c depicts the charge-discharge profiles of the LFP-2/C sample tested at a rate of 0.2 C (156 mAhg^−1^). It clearly shows that the sample's discharge capacity only decreased slightly, and 144 mAh g^−1^ can be maintained after 100 cycles. The hierarchically structured LFP-2/C exhibited an excellent high-rate capability, as presented in Fig. 5d. It clearly shows that there was no obvious loss of capacity at a rate as high as 20 C, with a capacity of 95% remaining even after 1000 cycles.

The rate capability of an insertion electrode material depends on the kinetics of the lithium ion extraction/insertion process. In the case of LFP, Li^+^ ion diffusion takes place along the *b*-axis in the crystal of LFP crystal during charging and discharging. Furthermore, charge transfer occurs mainly in the *ac*-plane. Therefore, the rate performance of LFP depends on the particle size and the crystal facet. Consequently, the crystal orientation of LFP has significant effects on its electrochemical reaction processes. The LFP-2 sample has larger facets in the *ac*-plane and is thinner in the *b*-direction than others, resulting in an increase in the electrochemical reaction surface area, and enhancement in electrical conductivity and Li^+^ diffusion. These improvements may indicate why the LFP-2/C sample exhibited excellent electrochemical properties.

## Discussion

A simple one-step solvothermal route was developed for synthesizing hierarchically structured LFP. These hierarchically structured LFP samples were constructed from nanostructured platelets with their (010) facets mainly exposed. Based on a series of experimental results, a tentative mechanism for the formation of these hierarchical structures was proposed. After these hierarchically structured LFP samples were coated with a thin carbon layer and used as cathode materials for LIBs, they exhibited excellent high-rate discharge capability and cycling stability. For instance, a capacity of 95% can be maintained for the LFP-2/C sample at a rate as high as 20 C, even after 1000 cycles. This behavior might be due to the unique hierarchical structure and crystal orientation of LFP, which both help to facilitate the fast and efficient transport of mass and charge, resulting in an excellent rate capability. Therefore, the present work might renew interest in the design of nanostructured cathode materials in the future.

## Methods

### Synthesis of ionic liquid (IL)

Choline chloride (ChCl) was recrystallized from absolute ethanol, and was then filtered and dried under vacuum. Ethylene glycol (EG) was used as a receiver. The 200 mL of solution was obtained by the mixture of ChCl and EG in a ratio of 1:2 at 75°C and stirred until a homogeneous colorless liquid was formed.

### Synthesis of LiFePO_4_

Hierarchically structured LiFePO_4_ was synthesized by a one-step solvothermal reaction at low temperature using ionic liquid (IL) as a solvent. For a typical process, firstly, IL was pre-heated to 50°C to insure the precursor to be well dissolved. Secondly, CH_3_COOLi, FeSO_4_·7H_2_O and NH_4_H_2_PO_4_ in a mole ratio of 1:1:1 as the precursors were added into 10 mL IL under vigorous stirring for 30 min. Then, the obtained homogeneous solution was transferred into a 20 mL Teflon-lined stainless steel autoclave, and heated at 200°C for 24 h. After cooled to room temperature, the green precipitate was washed thoroughly with water and ethanol for several times, and dried under vacuum at 60°C overnight. To investigate the process of LFP formation, the samples were synthesized with different concentrations of precursor (0.1, 0.2 and 0.4 mmol) and donated as LFP-1, LFP-2 and LFP-3.

### Synthesis of LiFePO_4_/C composite

To prepare LiFePO_4_/C composite, the as-prepared LiFePO_4_ and sucrose (10 wt%) were dispersed in distilled water and ethanol, and dispersed further by ultrasonication and concentrated to dryness. The mixture was milled in mortar and then calcined in Ar atmosphere at 300°C for 0.5 h, and then increased to 550°C for 2.5 h. The heating rate is 5°C min^−1^.

### Characterizations

Scanning electron microscopy (SEM, Hitachi S4800 instrument) and Transmission electron microscopy (TEM, FEI F20 S-TWIN instrument) were applied for the characterizations of crystal structure and morphology. X-ray diffraction (XRD) patterns were recorded on a PANalytical X'Pert spectrometer using the Co Kα radiation (λ = 1.78897 Å), and the data were changed to Cu Kα data.

### Electrochemical measurements

For the electrochemical measurement of Li-ion intercalation, LiFePO_4_/C composite were mixed with polyvinylidene fluoride (PVDF) binder and acetylene black carbon additive in a weight ratio of 70:20:10. The mixture was spread and pressed on aluminum foil circular flakes as working electrodes, and dried at 120°C in vacuum for 12 h. Lithium foils were used as the counter electrodes. The active material content in the electrode was around 1.5 mg. The electrolyte was 1 M LiPF_6_ in a 1/1/1 (volume ratio) mixture of ethylene carbonate (EC), ethylene methyl carbonate (EMC) and dimethyl carbonate (DMC). The separator was UP 3093 (Japan) micro-porous polypropylene membrane. The cells were assembled in a glove box filled with highly pure argon gas (O_2_ and H_2_O levels < 1 ppm), and charge/discharge tests were performed in the voltage range of 2 to 4.2 V at different current densities on a Land automatic batteries tester (Land CT 2001A, Wuhan, China). Cyclic voltammetry (CV) measurements of the cells were carried out using a CHI 660c electrochemical workstation.

## Author Contributions

B.B.G. and M.D.W. proposed and designed the experiments. B.B.G. and H.C.R. carried out the synthetic experiments and conducted the characterization. B.B.G., C.Z. and H.L.F. performed the HRTEM, SEM characterization and structural analysis. B.B.G. and M.D.W. analysed the data and wrote the manuscript. All the authors participated in discussions of the research.

## Supplementary Material

Supplementary Information Supporting Information
