# Supplementary material for: Hierarchical LiFePO4 with a controllable growth of the (010) facet for lithium-ion batteries
Source: Sci Rep. 2013 Sep 27;3:2788. doi: 10.1038/srep02788 (PMC3784946; doi:10.1038/srep02788)
Supplement: Supplementary Information — Supporting Information [file srep02788-s1.doc]

**Supporting Information**

**Hierarchicald LiFePO_4_ with a controllable growth of the (010) facet for lithium-ion batteries**

Binbin Guo, Hongcheng Ruan, Cheng Zheng, Hailong Fei, Mingdeng Wei*

*Institute of Advanced Energy Materials, Fuzhou University, Fuzhou, Fujian 350002, China; E-mail: wei-mingdeng@fzu.edu.cn


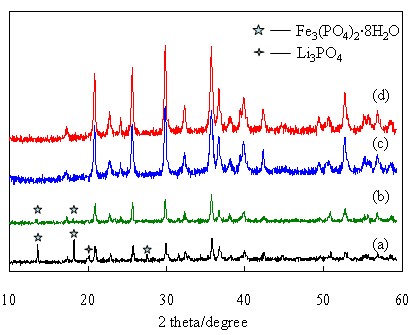


**Figure S1│ XRD patterns of the samples obtained at different reaction times:** (a) 6, (b) 12, (c) 18 and (d) 24 h.


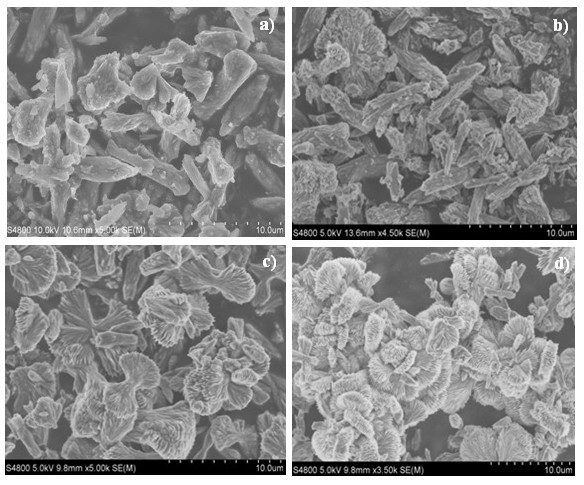


**Figure S2│ SEM images of the samples synthesized at different reaction times:** (a) 6, (b) 12, (c) 18 and (d) 24 h.


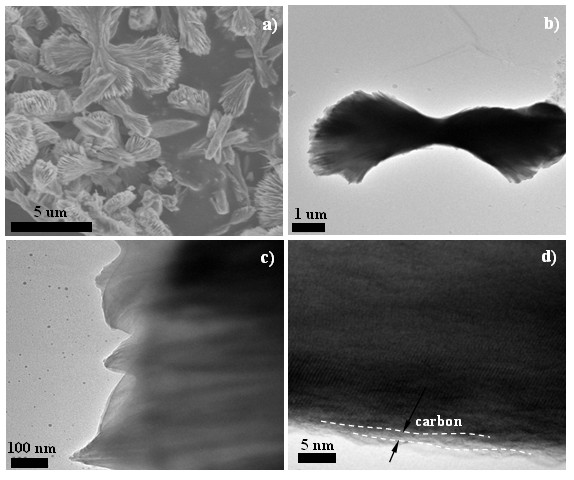


**Figure S3│** (a) SEM and (b-d) TEM images of the LFP-2 samples after carbon coating.

**
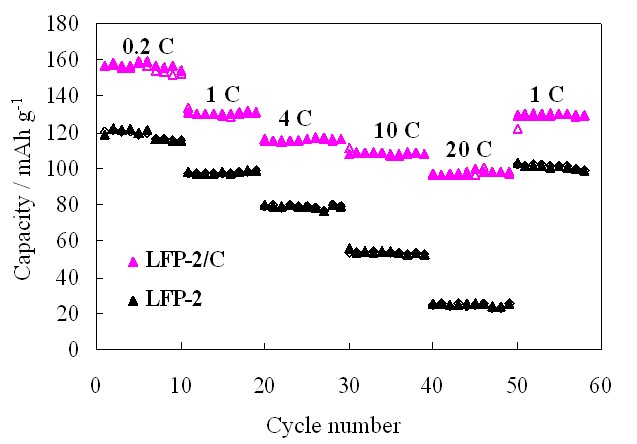
**

**Figure S4│** Rate performance of uncoated LFP-2 and carbon coating LFP-2/C composite.
